# Influence of the Change of Phase Composition of (1 − x)ZrO_2_–xAl_2_O_3_ Ceramics on the Resistance to Hydrogen Embrittlement

**DOI:** 10.3390/ma16227072

**Published:** 2023-11-07

**Authors:** Inesh E. Kenzhina, Artem L. Kozlovskiy, Meiram Begentayev, Aktolkyn Tolenova, Saulet Askerbekov

**Affiliations:** 1 Department of General Physics, Satbayev University, 22 Satbayev Street, Almaty 050013, Kazakhstankozlovskiy.a@inp.kz (A.L.K.);; 2 Institute of Experimental and Theoretical Physics, Al-Farabi Kazakh National University, 71 Al-Farabi Avenue, Almaty 050040, Kazakhstan; 3 Laboratory of Solid State Physics, The Institute of Nuclear Physics, Almaty 050032, Kazakhstan; 4 Laboratory of Advanced Electronics Development, Kazakh-British Technical University, 59 Tole Bi Street, Almaty 050000, Kazakhstan

**Keywords:** oxide ceramics, radiation damage, hydrogen embrittlement, swelling, thermo-physical properties

## Abstract

The article describes the influence of the change in the phase composition of ceramics on the stability of the crystal structure and retention of thermo-physical parameters during hydrogenation of the surface layer in the proton irradiation process. The selection of irradiation conditions allows modeling the degradation processes of ceramics associated with gas swelling during hydrogenation, as well as revealing the patterns of the effect of phase composition on embrittlement, de-strengthening, and structural degradation resistance. In the course of the conducted studies, dose-dependencies of irradiation-induced structural changes and consecutive accumulation of radiation-induced damage in ceramics as a result of hydrogenation of the damaged near-surface layer were established. It was found that the maximum structural changes are observed at doses above 10^15^ protons/cm^2^. Dependencies of the change in the degree of structural order as a function of the dose of accumulated damage and the concentration of accumulated protons were obtained. It was established that the variation of the ceramics phase composition due to the formation of solid solutions of ZrO_2_/Al_2_O_3_ and ZrO_2_/Al_2_O_3_/AlZr_3_ type leads to an enhancement of resistance to swelling by 3–5 times in comparison with monoclinic ZrO_2_ ceramics. The general analysis of the variation of strength and thermo-physical parameters of ceramics as a function of irradiation fluence for ceramics with different phase compositions showed a direct dependence of the decrease in hardness, resistance to cracking, and thermal conductivity on the concentration of deformation structural distortions caused by irradiation.

## 1. Introduction

One of the most attractive prospects for the advancement of nuclear industry, which is the most interesting sector for solving the disbalance in the global energy sector and filling the global energy deficit in the future, is the substitution of conventional uranium fuel with plutonium fuel, as well as the transition to the operation of high-temperature nuclear reactors capable of operating at elevated temperatures (above 600 K), as well as operating for a long time, maintaining the stability of the core [1,2]. As a rule, in modern reactors, one of the limiting factors for extended lifetime service is the low burnup factor of nuclear fuel, which is caused by a high degree of radiation embrittlement and destruction of fuel element materials, which are unable to withstand prolonged exposure to fission fragments and neutrons. As a solution to this problem, in the last few years, a variant of transition from classical uranium fuel in the form of fuel rods consisting of uranium dioxide pellets to dispersed fuel has been considered [1,2], which is rooted in the technology of placing fissile nuclear material (uranium dioxide or plutonium) in an inert matrix in oxide or nitride ceramics [3,4]. At the same time, most of the currently proposed technological solutions are based on the use of weapons-grade plutonium as fissile material, the application of which will solve a number of issues with its existing stockpiles as well as decrease the volume of nuclear waste. The interest in this line of research is conditioned by the increasing awareness of the global audience of the issue of handling spent nuclear fuel, particularly its subsequent use or disposal. Inert matrices based on ZrO_2_ ceramics doped with cerium, aluminum, and magnesium are considered alternative materials in new nuclear fuels [1,5,6,7]. Meanwhile, in the latest years, considerable importance has been given to the study of the radiation resistance of these ceramics, the knowledge of which will allow predicting the possibilities and areas of use of these types of ceramics as materials for the development of nuclear fuel. The interest in these studies lies primarily in the possibility of assessing the radiation resistance of the proposed candidate inert matrix materials under conditions as close as possible to real operating conditions, as well as establishing critical points at which it is impossible to use these ceramics as inert matrix materials in the future. Also an important factor in these studies is the possibility of expanding the fundamental understanding of the basis of interaction of ionizing radiation with ceramic materials, the mechanisms of which have certain features due to the dielectric nature of most oxide and nitride ceramics, as well as high melting temperatures, which leads to a reduction of factors related to the thermal effects of interaction of ionizing radiation with target materials [8,9,10,11]. At the same time, mechanisms of radiation damage accumulation and their subsequent evolution, which can lead to material destabilization, are quite different in accordance with the type of ionizing radiation interacting with the ceramic material. However, for heavy ions, these structural variations are usually associated with the occurrence of mechanical residual stresses leading to embrittlement of the surface layer and its partially exfoliating [12,13]. When interacting with helium or protons, the key role in destabilization of the damaged layer is played by the mechanisms of creating gas-filled inclusions in the form of bubbles or cavities, the increase in the concentration and size of which leads to swelling of the affected layer and subsequent explosive damage of the affected layer [14,15,16]. Moreover, the development of gas-filled regions as a consequence of hydrogenation and helium swelling can happen both in the accumulation of nuclear reaction products and during the interaction of the near-surface layer with the coolant [17,18].

In this work, the effects occurring in samples of (1 − x)ZrO_2_–xAl_2_O_3_ ceramics under irradiation with protons with an energy of 1.5 MeV and fluences from 1011 to 1017 protons/cm^2^ are considered. The choice of (1 − x)ZrO_2_–xAl_2_O_3_ ceramics samples with different phase compositions, the variation of which is caused by the use of different stoichiometric ratios of zirconium and aluminum oxide compounds, as objects of study is primarily conditioned by their advanced strength and thermo-physical parameters, the totality of which allows us to consider them as one of the most promising materials for the inert matrices of dispersed nuclear fuel for high-temperature nuclear reactors [19,20,21].

## 2. Materials and Methods

Micron-sized (2–3 µm) ZrO_2_ and Al_2_O_3_ powders acquired from Sigma Aldrich (St. Louis, MO, USA) were used for the synthesis of cer-cer ceramics based on ZrO_2_ compounds doped with aluminum oxide. The chemical purity of the powders used for synthesis was 99.95%. Synthesis of ceramics was performed by varying the composition components (1 − x)ZrO_2_–xAl_2_O_3_ in the range x = 0.1–0.5 M. The ceramics synthesis was implemented by applying the mechanical-chemical method, which consists of grinding the initial powders in a predetermined mole proportion in a planetary mill. A planetary mill, the PULVERISETTE 6 classic line (Fritsch, Berlin, Germany), was applied for grinding. An 80 mL tungsten carbide beaker was used as a grinding cup, the use of which is conditioned by the necessity to avoid impurities in the process of grinding mixtures with increased hardness. The ratio of the initial mixture to the grinding balls was 2:1. The choice of a beaker and grinding balls made of tungsten carbide was due to its high resistance to mechanical stirring as well as the absence of the likelihood of impurities getting into the resulting powders, which was confirmed by X-ray phase analysis by determining the absence of any impurities associated with tungsten. Grinding was realized for 30 min at a grinding speed of 250 rpm. The method of mechanochemical synthesis was used to mix the initial powders and bring them to a homogeneous composition and size of powder particles, which were subsequently annealed in order to initiate the process of phase transformations. The selection of mixing conditions was carried out in such a way that the processes of phase transformations during stirring were not initiated in the samples (low grinding speeds and short mechanical stirring times). After mixing, the average size of the resulting powders was about 1 ± 0.1 µm, while in the initial state they were used with a diameter of about 2–3 µm. Following grinding, the obtained powdered mixtures were then annealed in a muffle furnace at 1500 °C (heating rate 10 °C/min) for 8 h, followed by subsequent cooling with the furnace for 24 h. The samples were annealed in an inert atmosphere (argon) to reduce the effects of reverse polymorphic transformations. Refrigeration of ceramics for such a long time during cooling is due to the need to avoid the effects associated with rapid refrigeration, which can result in surface oxidation and the creation of additional defects in the structure with a sharp change in temperature. To conduct studies related to irradiation and subsequent measurements of hardness and thermal conductivity, the samples were pressed in the form of tablets with a diameter of 10 mm and a thickness of about 20 µm. Pressing was carried out at a pressure of 250 MPa for 1 h, followed by heat treatment at a temperature of 400 °C in order to relieve mechanical stresses associated with pressing processes. The porosity of the samples was determined using the gravimetric method.

The selection of protons with an energy of 1.5 MeV as ionizing radiation is conditioned by the possibilities of simulation of radiation-induced damage processes characteristic of the effect of hydrogenation of the damaged layer, comparable to the real processes of ceramic hydrogenation during operation during nuclear reactions of fissile nuclear fuel as well as the operation of ceramics in the core during their interaction with coolants. The hydrogenation effect and helium swelling are among the most dangerous types of radiation damage due to the high mobility and capability of hydrogen and helium agglomeration in voids, with the subsequent development of gas-filled cavities resulting in deformation, swelling, and destructive embrittlement of the affected layer.

The irradiation was carried out at the UKP-2.1 accelerator at the Institute of Nuclear Physics. In order to avoid the effects of local overheating of the samples while irradiating, the samples were positioned on dedicated water-cooled holders, the use of which allowed for a stable temperature of the target during the whole irradiation experiment.

The choice of irradiation doses from 1011 to 1017 protons/cm^2^ is explained by the possibility of simulation of the effects of implanted hydrogen accumulation in the ceramic composition with its subsequent agglomeration and the formation of gas-filled bubbles in the affected layer with a thickness of 10–15 μm. According to a number of literature data [22,23], the swelling effect is characteristic for irradiation fluences of the order of 10^16^–10^17^ protons/cm^2^, for which the concentration of implanted hydrogen, according to calculated data using the SRIM Pro 2013 software code v.5.0, can be of the order of 1–5% at the maximum.

The X-ray diffraction method was used as the main evaluation method in order to determine the changes in structural parameters and mechanisms of deformation distortions of the damaged layer, as well as changes in the degree of structural ordering (degree of crystallinity) depending on the irradiation fluence. The measurements were carried out on a D8 Advance ECO X-ray diffractometer (Bruker, Berlin, Germany), in Bragg-Brentano geometry in the angular range 2θ = 20–90°, with a step of 0.03°. To eliminate texturing effects, the samples were rotated during measurements at a speed of 15 rpm. The phase composition was refined using card values from the PDF-2 (2016) database. To determine the phase composition, a technique was used to analyze the weight contributions of diffraction reflections characteristic of the established phases, followed by calculating their values using the formula $V_{\mathrm{admixture}}=\frac{RI_{\mathrm{phase}}}{I_{\mathrm{admixture}}+RI_{phase}},$ where *I_phase_* is the average integrated intensity of the main phase of the diffraction line; *I_admixture_*—average integral intensity of the additional phase; *R*—structure coefficient equal to 1.45.

Measurements of strength properties, in particular hardness, crack resistance, resistance to one-time compression, and cracking, were performed according to the developed measurement regulations, taking into account state standards on testing machines. The obtained data made it possible to identify the dynamics of variation in the strength properties of ceramics as a consequence of the accumulation of radiation damage and the agglomeration of implanted hydrogen in the near-surface layer, as well as to define dependences between changes in structural parameters associated with deformation distortions and changes in strength properties.

The thermo-physical parameters were analyzed using the method of estimating the heat flux of ceramics before and after irradiation, which in turn made it possible to determine the kinetics of change in the thermal conductivity coefficient of ceramics depending on the irradiation fluence and the degree of structural damage to ceramics. Assessment of the change in the thermal conductivity coefficient was carried out by means of a comparative analysis of the obtained values of irradiated samples with the data of the initial samples in the unirradiated state. In this case, the phonon mechanism of heat transfer in ceramics was considered the main mechanism of heat transfer in the case of dielectric ceramics.

## 3. Results and Discussion

### 3.1. Phase Composition Parameterization of Investigated Samples of (1 − x)ZrO_2_–xAl_2_O_3_ Ceramics at Changing Components

**Figure 1:**
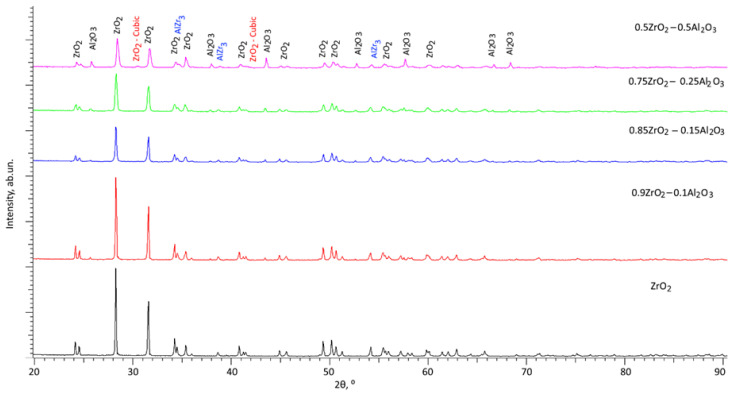
X-ray diffraction patterns of the investigated samples of (1 − x)ZrO_2_–xAl_2_O_3_ ceramics.

Figure 1 illustrates the phase analysis results of the investigated ceramics in relation to the ratio of synthesized components. The X-ray diffractogram of ZrO_2_ annealed at 1500 °C under the same conditions as the mixed composition samples is presented as a comparison sample.

The analysis of the ZrO_2_ sample chosen as a sample for comparison after thermal annealing showed that annealing of ZrO_2_ ceramics at a temperature of 1500 °C (0.55Tmelt) does not lead to phase polymorphic transformations, and the occurrence of any reflexes that are characteristic of impurity phases was not found on the diffractogram. The obtained data indicate that pure ZrO_2_ ceramics retain the monoclinic structure (space group P121/c1(14)) at an annealing temperature equal to half of the melting temperature.

Analysis of the presented X-ray diffractogram of the sample containing 0.1 mol of Al_2_O_3_ showed that the main reflexes are characteristic of the monoclinic phase of ZrO_2_. However, the observed reflections at 2θ = 25.653°, 35.372°, 52.992°, and 57.548° are characteristic of the Al_2_O_3_ phase (spatial syngony R-3c(167)) with a hexagonal type of crystal lattice. The presence of these reflexes, as well as their shape, indicates that the process of mechanochemical synthesis and then thermal annealing results in the development of a structure containing inclusions in the form of a solid solution of Al_2_O_3_ without the formation of complex oxides or spinel-type structures. The assessment of the area contribution of the reflections indicates that the ratio of ZrO_2_: Al_2_O_3_ phases for the studied sample was 89: 11%, which in general has a good agreement with the molar ratio of the initial components used for synthesis.

By analyzing the resulting X-ray diffractogram of the 0.75ZrO_2_–0.25Al_2_O_3_ ceramic sample, it was shown that the input of hexagonal Al_2_O_3_ phase grows up to 30% and the input of cubic AlZr_3_ phase increases up to 5%, which indicates that the content of impurity inclusions becomes higher. The growth of the contribution of additional phases results in the formation of additional interphase boundaries, the presence of which determines the strength properties of ceramics as well as their resistance to external effects.

In the case where the ratio of ZrO_2_ and Al_2_O_3_ components becomes equal during synthesis, the phase composition evaluation showed that the content of the monoclinic ZrO_2_ phase is not more than 58–60% and the content of the Al_2_O_3_ phase is more than 35%. In addition to the previously detected phases Al_2_O_3_ and AlZr_3_ in the ceramics, the diffraction reflections characteristic of the cubic phase ZrO_2_ (space group Fm-3m(225)) are observed on the diffractograms obtained, the formation of which, in accordance with the received data, indicates the occurrence of polymorphic transformations of the type m-ZrO2 → c-ZrO2. Polymorphic transformations of this type in ZrO_2_ can result in its hardening and increased resistance to external influences and mechanical stresses.

Thus, summarizing the characterization of changes in the phase composition of (1 − x)ZrO_2_–xAl_2_O_3_ ceramics at variation of weight contributions of the components, the general dynamics of phase transformations can be described in the following format: ZrO_2_ → ZrO_2_/Al_2_O_3_ → ZrO_2_/Al_2_O_3_/AlZr_3_ → ZrO_2_/Al_2_O_3_/AlZr_3_/c-ZrO_2_.

### 3.2. Study of Structural Deformations in (1 − x)ZrO_2_–xAl_2_O_3_ Ceramics Caused by Proton Irradiation

One of the ways to evaluate the influence of radiation damage on the properties of structural materials or ceramics is through the application of the X-ray diffraction method used to define the kinetics of changes in structural parameters and their deformation as a result of accumulating radiation damage. In addition, a number of studies have established that deformation distortions of the crystal structure of ceramics as a result of radiation damage accumulation can lead to the complete destability of ceramics and their amorphization [24,25]. Also, the accumulation of structural changes associated with radiation damage for zirconium dioxide-based ceramics can initiate the processes of polymorphic transformations, which are followed by degradation of both strength and thermo-physical parameters. Using X-ray diffraction data, in particular, the determination of changes in the characteristics of the crystal lattice and its volume, it is possible to define the influence of irradiation on their changes, as well as to find out the deformation distortion type (compressive or tensile) arising from the accumulation of structurally altered regions in the damaged layer.

**Figure 2:**
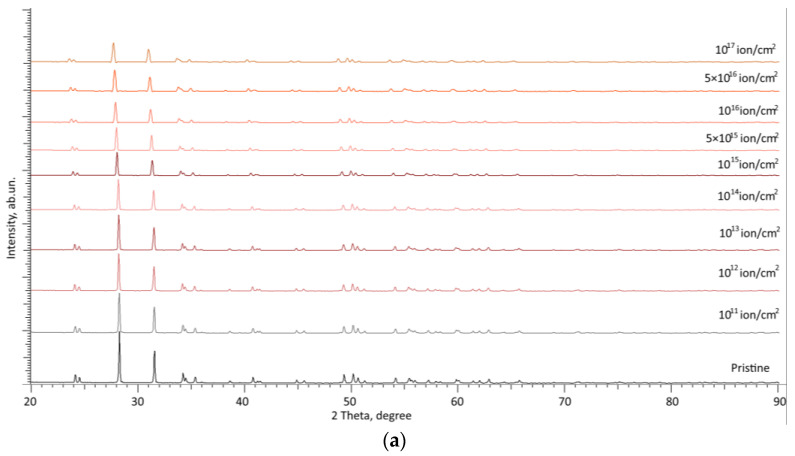

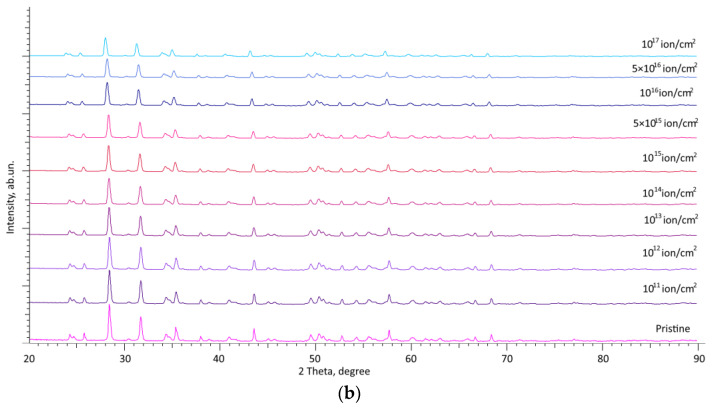
Results of X-ray diffraction of the studied ZrO_2_ (**a**) and 0.5ZrO_2_–0.5Al_2_O_3_ (**b**) ceramics exposed to irradiation by protons with different irradiation fluences.

Figure 2 demonstrates the results of X-ray diffraction data for two samples of ZrO_2_ and 0.5ZrO_2_–0.5Al_2_O_3_ ceramics, presented in order to reflect the dynamics of changes in the structure of ceramics with increasing irradiation fluence. These samples of ZrO_2_ and 0.5ZrO_2_–0.5Al_2_O_3_ ceramics were selected as extreme cases in order to determine the effect of the addition of aluminum oxide to the composition of zirconium dioxide-based ceramics on their stability and growth in resistance to structural disorder. The measurements were carried out according to the following scheme: The sample was subjected to X-ray diffraction in its initial state as well as the determination of its structural parameters, which were recorded as initial values (the Pristine diffraction pattern of the sample). Subsequently, this sample was irradiated until a certain fluence was achieved and was again subjected to radiography as well as measurement of structural parameters, which were compared with the original sample in order to determine the contribution of structural distortions and swelling resulting from irradiation. After all the necessary measurements, the sample was irradiated to the next fluence value, taking into account the previously accumulated radiation dose, and all measurements were repeated again in order to determine the dynamics of changes in structural parameters depending on the irradiation fluence. Effects associated with the possible relaxation of structural distortions caused by irradiation were taken into account when assessing the structural parameters, since the relaxation effect manifests itself only at low irradiation fluences, which are characterized by high isolation of structural distortions in the damaged ceramic layer. In the case of high-dose radiation, this effect is minimal.

As can be seen from the presented set of X-ray diffraction patterns, the main changes that occur with increasing irradiation fluence are associated with broadening of reflections as well as a shift of diffraction maxima to the region of small angles, which indicates deformation distortion of the crystal structure. At the same time, in the case of both types of ceramics, the appearance of new diffraction reflections was not established, which indicates the absence of phase or polymorphic transformation processes caused by irradiation. In turn, structural distortions are more pronounced for ZrO_2_ ceramics, while for 0.5ZrO_2_–0.5Al_2_O_3_ ceramics, the alteration in diffraction patterns is less pronounced, which indicates elevated stability to radiation damage caused by the accumulation of structural distortions.

**Figure 3:**
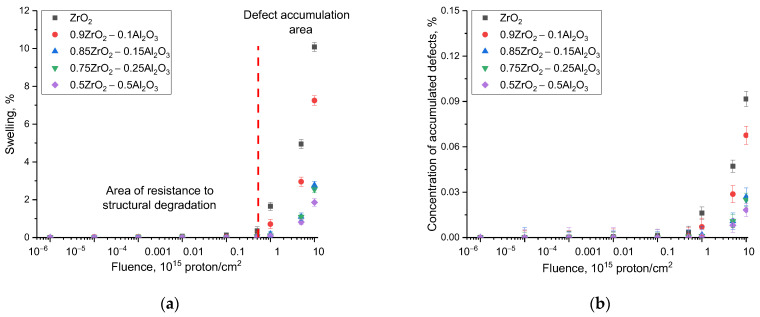
Graphs of structural distortions: (**a**) dependence of crystal lattice swelling on irradiation fluence; (**b**) dependence of the concentration change of the defective fraction on the irradiation fluence.

Figure 3 demonstrates the estimation results of the lattice swelling value characterizing the deformation distortion, as well as the dependence of the change in the concentration of the defective fraction in the damaged layer as a function of the irradiation fluence. The estimation of the lattice swelling was based on Formula (1):

(1) $$Swelling=\left(\frac{V-V_{0}}{V_{0}}\right)\times 100\%$$

where *V* and *V*_0_ are the volumes of the crystal lattice for the irradiated and original samples. The swelling results were calculated by a comparative analysis of changes in the parameters and volume of the crystal lattice for each established phase, followed by calculating the average value. Formula (2) was used to calculate the concentration of the defective fraction based on the intensity changes of diffraction reflections and their area.

(2) $$f_{A}=1-\frac{\sum_{n}^{i=1}\frac{A_{i}^{irrad}}{A_{i}^{unirrad}}}{n},$$

where $A_{i}^{irrad}$ and $A_{i}^{unirrad}$ are the contributions of diffraction reflections before and after irradiation, and ***n*** is the number of diffraction reflections.

It should be noted that in accordance with the data of X-ray phase analysis in the whole measured range of irradiation fluences for all studied samples of (1 − x)ZrO_2_–xAl_2_O_3_ ceramics, changes related to polymorphic phase transformations typical for zirconium dioxide with a monoclinic type of crystal structure were not found, which indicates that at proton irradiation, the values of ionization losses of protons with an energy of 1.5 MeV are not sufficient for the initialization of phase transformation processes.

The main changes in the structural characteristics, in particular the degree of swelling and growth of the defect fraction concentration, occur at fluences higher than 1015 proton/cm^2^, for which the change in parameters evidences the accumulation of tensile-type strain distortions in the structure. At irradiation doses below 1015 proton/cm^2^, the main structural changes are connected with the formation of locally isolated structurally distorted regions in the damaged layer of ceramics, the accumulation of which is accompanied by a small growth of the contribution of tensile strain distortions in the crystal structure. Such large differences in the change in structural disorder trends for ceramics irradiated with protons in comparison with the results of experimental studies [26,27,28,29,30] devoted to the irradiation of ceramics with heavy ions are due to several factors. Firstly, when irradiated with heavy ions, due to their size and energy, the radiation damage degree quickly reaches a maximum concentration that can lead to destruction of the crystal structure, as well as the effects of amorphization and embrittlement. As a rule, these effects for high-energy ions are observed at fluences above 10^12^–10^13^ ions/cm^2^. Here, when irradiated with heavy ions (even in the case of energies above 200 MeV), the maximum thickness of the affected layer is no more than 20–25 μm [31,32].

**Figure 4:**
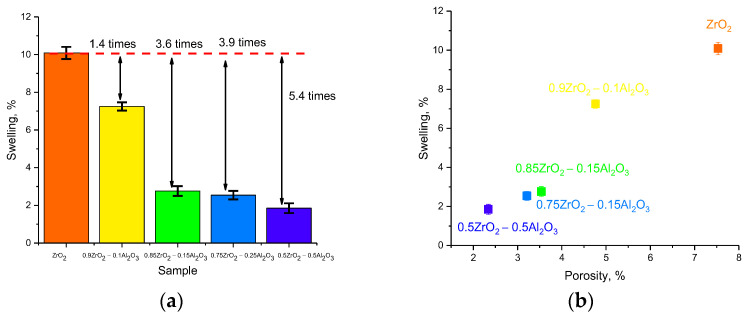
(**a**) Results of comparison of the increase in swelling resistance of ceramics compared to ZrO_2_ ceramics; (**b**) Comparative analysis of changes in the maximum value of swelling and porosity of ceramics in the initial state, determined using the gravimetric method.

With proton irradiation, due to the small size of the protons themselves as well as their high penetration ability, ionization losses of protons in interaction with the crystal structure have a much smaller effect than heavy ions, and the main structural damage is primarily due to the accumulation effect associated with the agglomeration of implanted hydrogen with the subsequent formation of gas-filled bubbles [33,34]. The formation of such inclusions, as a rule, occurs at high irradiation fluences (above 1016 proton/cm^2^), for which the calculated concentration of implanted hydrogen can be several atomic percent. In this regard, the observed kinetics of the change in structural parameters have good correspondence with this substantiation, since the most pronounced changes occur at fluences above 1015 proton/cm^2^. In such a case, the greatest structural changes caused by degradation and swelling of the crystal lattice due to the accumulation of structurally deformed regions as well as the formation of gas-filled regions are observed for ZrO_2_ ceramics. In the case of two- to three-phase ceramics, the effects relating to deformation and swelling of the crystalline structure are less pronounced because of the presence of interphase boundaries that reduce the possibility of agglomeration of implanted hydrogen in the affected layer of ceramics. As is evident from the reported data in Figure 4a, reflecting the value of stability of the crystal structure to swelling at the maximum irradiation fluence (1017 protons/cm^2^) in comparison with ZrO_2_ ceramics, the presence of Al2O3, AlZr3, and c-ZrO_2_ phases results in a significant increase in the stability of the crystal structure to radiation-induced swelling.

This phenomenon might also be accounted for by the presence of an increase in dislocation density and interfacial boundaries in the structure during the formation of two- to three-phase ceramics, the presence of which can prevent the agglomeration of implanted hydrogen by inhibiting it at the boundaries, preventing it from agglomerating into large gas-filled bubbles, leading to an increase in the deformation distortion of the crystal structure. According to the obtained data, the improvement of resistance to swelling with increasing concentrations of Al_2_O_3_ in the composition of ceramics results in a 3–5-fold increase in resistance to structural degradation in comparison with ZrO_2_ ceramics with a monoclinic type of crystal structure.

Figure 4b reveals the results of a comparative analysis of variations in the values of maximum swelling at maximum irradiation fluence and ceramic porosity, determined using the gravimetric method in the initial state. In the case of ZrO_2_ ceramics, the porosity value is maximum, which in turn leads to the formation of cavities in which hydrogen can accumulate, which leads to a sharp swelling of the ceramics. In the case when the composition of ceramics consists of two or more phases, the porosity of the samples decreases, which has a good correlation with the swelling values of ceramics during irradiation.

### 3.3. Investigation of Strength and Thermo-Physical Parameters Changes of (1 − x)ZrO_2_–xAl_2_O_3_ Ceramics in Dependence on Irradiation Fluence

**Figure 5:**
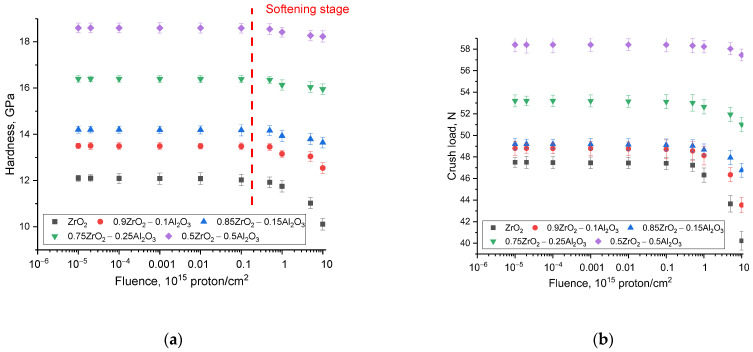
The evaluation of strength characteristics of (1 − x)ZrO_2_–xAl_2_O_3_ ceramics: (**a**) hardness change data; (**b**) data of changes in resistance to single compression.

Figure 5 summarizes the measured data on the strength characteristics (hardness and resistance of ceramics to single compression) as a function of irradiation fluence for all the samples studied. Received dependences on changes in strength properties were characterized in two stages. First, the influence of variation in the composition of (1 − x)ZrO_2_–xAl_2_O_3_ ceramics on the change in strength characteristics was determined in order to identify strengthening factors related to the change in phase composition. Secondly, the dependences of change in strength properties for irradiated samples at variation of irradiation fluence were determined. According to the data obtained for the initial samples, the change in phase composition increases the resistance of ceramics to both single compression and strengthening by increasing the hardness and resistance to cracking. In such a case, the increase in stability plays a very significant role in increasing the stability of the strength properties against radiation damage resulting from high-dose irradiation and subsequent swelling processes.

**Figure 6:**
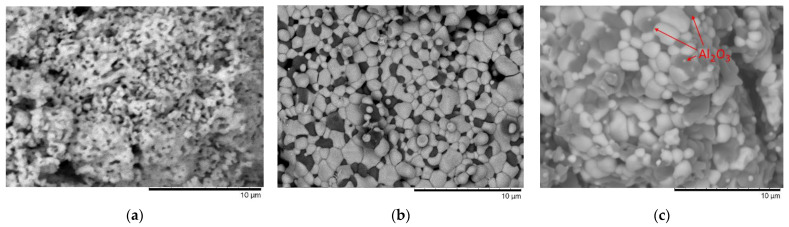

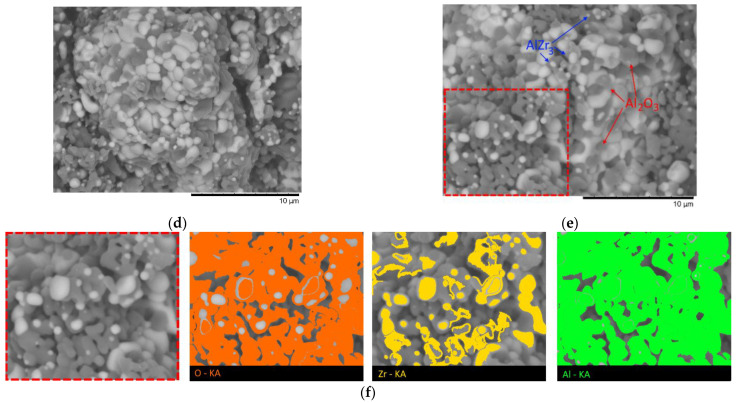
The results of changes in the morphological features of (1 − x)ZrO_2_–xAl_2_O_3_ ceramics with varying components: (**a**) ZrO_2_; (**b**) 0.9ZrO_2_–0.1Al_2_O_3_; (**c**) 0.85ZrO_2_–0.15Al_2_O_3_; (**d**) 0.75ZrO_2_–0.25Al_2_O_3_; (**e**) 0.5ZrO_2_–0.5Al_2_O_3_. (**f**) Mapping results of the 0.5ZrO_2_–0.5Al_2_O_3_ ceramic sample, reflecting the presence of AlZr_3_ grains.

It has been found that the destruction of the near-surface layer leads to disorder in the damaged layer, resulting in a decrease in the thermo-physical and strength properties of the ceramic. It was found that the occurrence of inclusions in the form of AlZr_3_ solid solutions in the composition of ceramics increases the resistance to embrittlement and softening by 1.5–2 times in comparison with ceramics based on zirconium dioxide under high-dose irradiation. This effect can be accounted for by the creation of additional interfacial boundaries that prevent the implanted hydrogen from clustering into gas-filled blisters in the damaged layer, resulting in an increase in resistance to embrittlement. Similarly, the strengthening of ceramics due to inclusions in the form of interstitial phases can also be explained by changes in the dislocation density of ceramics in the case of the occurrence of grains of different phases, the presence of which was established by means of the method of scanning electron microscopy (see the data presented in Figure 6). Identification of grains with different phases, as well as their determination, was carried out using the method of energy dispersive analysis by assessing the elemental composition at various points of the samples, as well as performing mapping and subsequent comparison of the elemental ratio data in these grains with the results of X-ray phase analysis (see data in Figure 6f). At the same time, no significant changes in the morphology of grains were observed after irradiation, due to small structural changes occurring inside the grains.

The evaluation of the variation of the parameters of crack resistance and resistance to single compression as a function of the irradiation fluence also indicates a positive impact of the presence of interfacial boundaries for the ceramics samples with an Al_2_O_3_ content higher than 0.15 M. In this case, a modification of the phase composition of the ceramics leads to an improvement of the resistance to destruction and preservation of the stability of the strength properties when irradiated with protons up to a dose of 5 × 10^16^ proton/cm^2^, while a reduction in crack resistance and an increase in softening factor are observed for ZrO_2_ ceramics at a fluence of 10^15^ proton/cm^2^.

**Figure 7:**
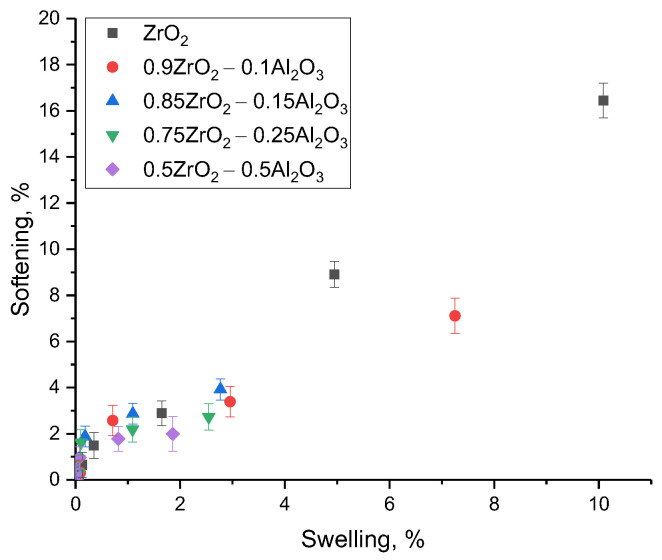
Results of a comparison analysis of the swelling and softening of ceramics as a result of irradiation.

Figure 7 shows the changes in the factors of reduction of the strength properties as a function of the degree of swelling in the ceramic composition. As shown by the dependencies of the change in the degree of softening on the degree of swelling in the composition of the affected layer, in the case of small deformations of the crystal structure, the softening does not exceed 5%, which is within the permissible operating limits. In this case, an increase in the extent of disorder due to deformation distortion of the crystal structure results in a sharp deterioration of the strength properties, which may cause destabilization of the resistance of the affected layer to mechanical stress. The presented dependence clearly shows a direct correlation between the swelling of the crystal structure due to its deformation and the softening of the damaged layer and the decrease in resistance to mechanical stress.

**Figure 8:**
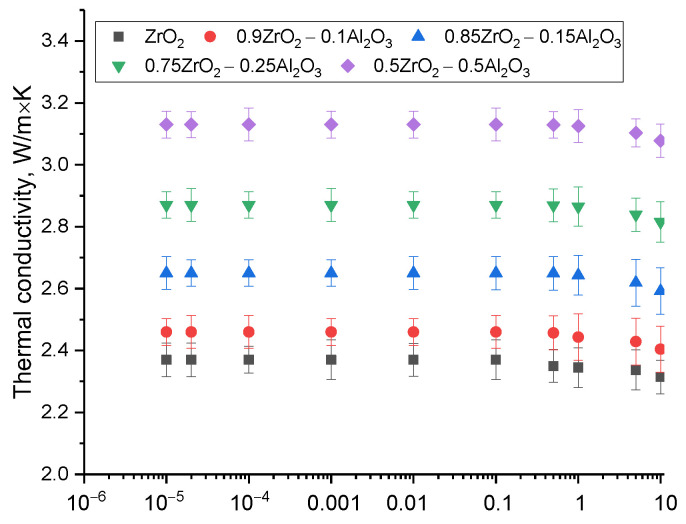
Evaluation results of the thermal conductivity variation of (1 − x)ZrO_2_–xAl_2_O_3_ ceramics.

Figure 8 presents the results of the evaluation of the thermal conductivity variation of ceramics with different phase compositions and irradiation fluence. The obtained dependences reflect the influence of various factors (changes in the phase composition of ceramics and irradiation fluence) on the change of thermo-physical parameters, which allowed to define the kinetics of changes in the thermal conductivity of ceramics on the concentration of defective inclusions.

An evaluation of the variation of the thermo-physical parameters of the ceramics as a function of the change in the phase composition indicates that the formation of two- or three-phase ceramics causes an increase in thermal conductivity of 30–50% compared to monoclinic ZrO_2_, which is due to the higher thermal conductivity of alumina, playing an essential part in the variation of the thermo-physical parameters of the ceramics. For irradiated samples, it has been found that the existence of interfacial boundaries, which increases stability and resistance to radiation-induced degradation, also has a positive effect on the preservation of thermo-physical parameters and the reduction of heat losses at high doses of irradiation. In the present case, a small degree of deformation does not have a significant effect on the phonon mechanisms of heat transfer, which results in the preservation of the thermo-physical parameters. Irradiation of ZrO_2_ ceramics with fluences above 10^15^ proton/cm^2^ decreases the thermal conductivity, which is mainly attributed to an increase in the concentration of deformation inclusions in the affected layer, which can serve as additional scattering centers in the phonon mechanism of heat transfer, reducing the heat transfer rate and increasing the heat losses in ceramics. Such an effect of reduction of thermal conductivity with increasing deformation distortions in ceramic structure may result in the appearance of local areas of overheating, which in the future may negatively influence the ceramic stability. A general analysis of the variation of the thermo-physical parameters of ceramics as a function of the irradiation fluence for ceramics with different phase compositions showed a direct relationship between the deterioration of the thermal conductivity and the concentration of deformation distortions in the structure induced by irradiation. In such a case, the maximum decrease in thermal conductivity is not greater than 2.5%, which suggests a sufficiently high resistance of ceramics to a decrease in thermo-physical parameters caused by high-dose proton irradiation.

## 4. Conclusions

On the basis of the data of the X-ray phase analysis of the original samples, it was determined that the variation of the ratio of the components of (1 − x)ZrO_2_–xAl_2_O_3_ leads to the formation of solid solutions of two phases at low content of Al_2_O_3_, and in the case of an equal ratio of the ZrO_2_ and Al_2_O_3_ components, the formation of an interstitial solid solution phase of the AlZr_3_ type is determined in the composition of ceramics, the content of which amounts to at least 5%. The formation of the AlZr_3_ phase at low concentrations is also observed at an Al_2_O_3_ content of 0.15–0.25 M; however, the weight contribution of these inclusions does not exceed 1–2%. At the same time, an analysis of the level of structural ordering as a function of the change in phase composition showed that the formation of two or three phases in the structure due to an increase in the concentration of Al_2_O_3_ leads to a decrease in the parameters of the crystal lattice, indicating an enhancement of the degree of crystallinity and ordering of the crystal lattice.

As a result of the conducted studies, it can be stated that the use of two-phase ceramics with a ZrO_2_–Al_2_O_3_ base is much more promising than ZrO_2_ ceramics, which, as shown by the experiments, are subject to structural degradation under high-dose proton irradiation, which simulates the hydrogenation processes in the ceramic near-surface layer. However, the key mechanism for enhancing the resistance to degradation induced by radiation for two- and three-phase ceramics is the influence of the occurrence of additional interfacial boundaries and a higher dislocation density, which results in a decrease in the possibility of implanted hydrogen agglomeration and the generation of gas-filled bubbles. The data obtained are in reasonable agreement with a series of previous studies that have hypothesized the dislocation and interfacial hardening of ceramics against radiation damage.

## Data Availability

Not applicable.
